# The association between diverse psychological protocols and the efficacy of psilocybin-assisted therapy for clinical depressive symptoms: a Bayesian meta-analysis

**DOI:** 10.3389/fpsyt.2024.1439347

**Published:** 2024-08-13

**Authors:** Mu-Hong Chen, Shu-Li Cheng, Yu-Chen Kao, Ping-Tao Tseng, Chih-Wei Hsu, Chia-Ling Yu, Fu-Chi Yang, Trevor Thompson, Tien-Wei Hsu, Chih-Sung Liang

**Affiliations:** ^1^ Department of Psychiatry, Taipei Veteran General Hospital, Taipei, Taiwan; ^2^ Division of Psychiatry, School of Medicine, College of Medicine, National Yang Ming Chiao Tung University, Taipei, Taiwan; ^3^ Department of Nursing, Mackay Medical College, Taipei, Taiwan; ^4^ Department of Psychiatry, Tri-Service General Hospital, National Defense Medical Centre, Taipei, Taiwan; ^5^ Department of Psychiatry, Beitou Branch, Tri-Service General Hospital, Taipei, Taiwan; ^6^ Institute of Biomedical Sciences, National Sun Yat-sen University, Kaohsiung, Taiwan; ^7^ Department of Psychology, College of Medical and Health Science, Asia University, Taichung, Taiwan; ^8^ Prospect Clinic for Otorhinolaryngology & Neurology, Kaohsiung, Taiwan; ^9^ Institute of Precision Medicine, National Sun Yat-sen University, Kaohsiung, Taiwan; ^10^ Department of Psychiatry, Kaohsiung Chang Gung Memorial Hospital, Kaohsiung, Taiwan; ^11^ Department of Pharmacy, Chang Gung Memorial Hospital Linkou, Taipei, Taiwan; ^12^ Department of Neurology, Tri-Service General Hospital, National Defense Medical Centre, Taipei, Taiwan; ^13^ Centre for Chronic Illness and Ageing, University of Greenwich, London, United Kingdom; ^14^ Department of Psychiatry, E-DA Dachang Hospital, I-Shou University, Kaohsiung, Taiwan; ^15^ Department of Psychiatry, E-DA Hospital, I-Shou University, Kaohsiung, Taiwan; ^16^ Graduate Institute of Clinical Medicine, College of Medicine, Kaohsiung Medical University, Kaohsiung, Taiwan

**Keywords:** psilocybin, psilocybin-assisted therapy, Bayesian meta-analysis, depressive symptoms, psychotherapy outcome research

## Abstract

**Objective:**

Psilocybin-assisted therapy has shown promising efficacy on clinical depressive symptoms. However, diverse psychological support or psychotherapy was performed with psilocybin treatment. This study aimed to explore the association of psychological protocols with the efficacy of psilocybin-assisted therapy for depressive symptoms.

**Method:**

Five major databases were systemic searched for clinical trials addressing psilocybin-assisted therapy for patients with clinical depressive symptoms. A Bayesian random-effects meta-analysis and meta-regression were performed. The effect size was mean difference (with 95% credible interval) measured by 17-Item Hamilton Depression Rating Scale.

**Results:**

There were 10 eligible studies including 515 adult patients with clinically diagnosed depression. The psychological protocols could be categorized into four types: (i) manualized directive psychotherapy(k=1); (ii) manualized nondirective psychological support(k=3), (iii) non-manualized nondirective psychological support(k=5); and (iv) non-manualized supportive psychotherapy(k=1). The pooled standard mean difference of psilocybin-assisted therapy was 10.08 (5.03-14.70).

**Conclusion:**

Compared with manualized nondirective psychological support, the other three psychological approaches did not differ significantly. The improvement of depressive symptoms was not associated with the psychological protocols in adult patients receiving psilocybin-assisted therapy.

**Systemic review registration:**

Open Science Framework: identifier (osf.io/3YUDV).

## Introduction

Major Depressive Disorder (MDD), a complex and deliberating mental disorder, differs significantly from typical transient sadness or the misery experienced in adverse circumstances (1). Substantial evidence indicates that MDD has recurrent or prolonged impacts on various domains, including activities of daily living, quality of life, cognitive function, and employment status. According to the report of World Health Organization, depression is a leading cause of disability worldwide and a major contributor to the overall global burden of disease (2). However, the treatment of MDD is suboptimal, and a third to half of people with MDD do not achieve adequate treatment response with multiple antidepressant trials (1). This underscores the demand for innovative therapeutic strategies with rapid and sustained antidepressant effects.

In recent decades, psychedelic medicine has been advancing, revealing promising evidence for the treatment of various psychiatric disorders. Psilocybin, a classic psychedelic, when paired with psychological support or psychotherapy, has demonstrated rapid and enduring antidepressant effects in several clinical trials (3–6). In 2018, the US Food and Drug Administration (FDA) awarded two breakthrough therapy designations for psilocybin in the treatment of treatment-resistant depression (TRD), followed by a designation for MDD in 2019 (7). On July 1, 2023, Australia has approved psilocybin for the treatment of depression (8). The first country classifies psychedelics as medicines at a national level.

The resurgent exploration of psilocybin for MDD management has sparked enthusiasm. In standard clinical trials, psilocybin is administered with accompanying psychological support or psychotherapy. The efficacy of psilocybin-assisted therapy in MDD is conceptualized as a synergistic interplay between psychotherapeutic techniques and psilocybin. However, the psychotherapeutic approaches employed in previous clinical trials vary widely (9). For example, the study by Carhart-Harris et al. conducted nondirective (or client-led) psychological support without specified manual (3), while another study performed directive psychotherapy with selecting Acceptance and Commitment Therapy (10). The absence of a standardized psychological support or psychotherapy poses challenges in training therapists in the best way to conduct psilocybin-assisted therapy, thereby limiting the application of RCT findings in clinical practice.

Pharmaceutical companies have significantly invested in clinical research and filed patents on the production of psilocybin as well as the therapeutic processes (e.g., training of therapists). In psilocybin clinical trials, the inclusion of psychological support or psychotherapy is expected to influence future labeling. The aim of the current study was to explore the association between psychological approaches and antidepressant efficacy in adult patients receiving psilocybin-assisted therapy. Our paper will provide important information for further studies to select the optimal psychological approaches is psilocybin-assisted therapy for adult patients with clinically diagnosed depression.

## Methods

The study protocol was registered with PROSPERO (521995). We followed the Preferred Reporting Items for Systematic Reviews and Meta analyses (PRISMA) (11), which can be found in **Appendix 1**.

### Data sources and search

Two reviewers independently searched MEDLINE, Cochrane Central Register of Controlled Trials (CENTRAL), EMBASE, ClinicalTrial.gov, and PubMed without language restrictions from database inception to January 15, 2024. We also searched grey literature and reviewed reference lists of included studies and related systematic reviews (12). Two authors independently screened and selected the studies, and discrepancies were resolved by consulting a third author. **Appendix 2** demonstrates the complete search strategies, and **Appendix 3** shows the reasons for exclusion.

### Eligibility criteria

Eligible studies were clinical trials in adult patients (≥18 years) with clinical depression (i.e., diagnosed depression), such as distress related to life-threatening diagnoses and terminal illness, MDD, or other psychiatric disorders with comorbid clinical depression. We considered clinical trials with the following study designs: blinded randomized controlled trials (RCTs), open-label RCTs, and single-arm pre-post studies. We excluded microdosing studies, follow-up studies (follow-up after trial completion), and studies with healthy volunteers. We also excluded conference abstracts, editorials, reviews, meta-analyses, case reports, and case series, as well as publications reporting duplicate data.

### Definitions and data extraction

The primary outcome was change in depressive symptoms from baseline (continuous outcome), as measured by a validated rating scale such as the Hamilton Rating Scale for Depression. When multiple measurement tools were used, they were selected in the following order: the Hamilton Rating Scale for Depression, Montgomery-Åsberg Depression Rating Scale, and Beck Depression Inventory (second edition). Regarding the classifications of psychotherapy, they are primarily based on the descriptions in the methods or e-methods sections of the clinical trial studies we included, as well as on whether the psychotherapy is manualized or directive. Outcome data were extracted from the original intention-to-treat analysis. Two authors independently extracted and reviewed the data, which was further verified by another author. WebPlot Digitizer (https://automeris.io/WebPlotDigitizer/) was used to extract numerical data from the figures. Two reviewers independently extracted data and discrepancies were resolved by consensus and, when necessary, by consulting corresponding authors. We extracted the methodology of the psychological support or psychotherapy of each trial. The extracted study-level variables include author, year, condition, psychotherapeutic model, therapist, preparation session, and integration session. We also extracted sample size, study design, dose of psilocybin, age, sex, comorbidities, and baseline and post-treatment severity of depressive symptoms.

### Risk of bias assessment

Two authors independently used the Cochrane randomized trial Risk of Bias tool (version 2.0) to assess the risk of bias in the included trials (13). Conflicts resolved through discussion with a third reviewer.

### Statistical analysis

All statistical analyses were performed using R version 4.3.1 (The R Foundation for Statistical Computing; Vienna, Austria). The meta-analysis within a Bayesian framework was fitted using the Bayesian statistical software Stan called multinma (14). The posterior distributions were obtained from tour Markov chains. There were 50000 iterations per chain and the first 20000 iterations for each chain were discarded as a warm-up. Convergence was assessed by visual inspection of the trace plots of the key parameters for each analysis. The prior settings and convergence results are shown in **Appendix 4**. The funnel plots and the test for funnel plot asymmetry were conducted using the R package metafor.

## Results

A total of 821 papers were identified in the search. After removing duplicates and excluding papers by title and abstract, 24 papers were considered for full-text review. Then,10 were considered eligible (**Supplementary Figure 1**).

### Demographic characteristics

**Figure 1:**
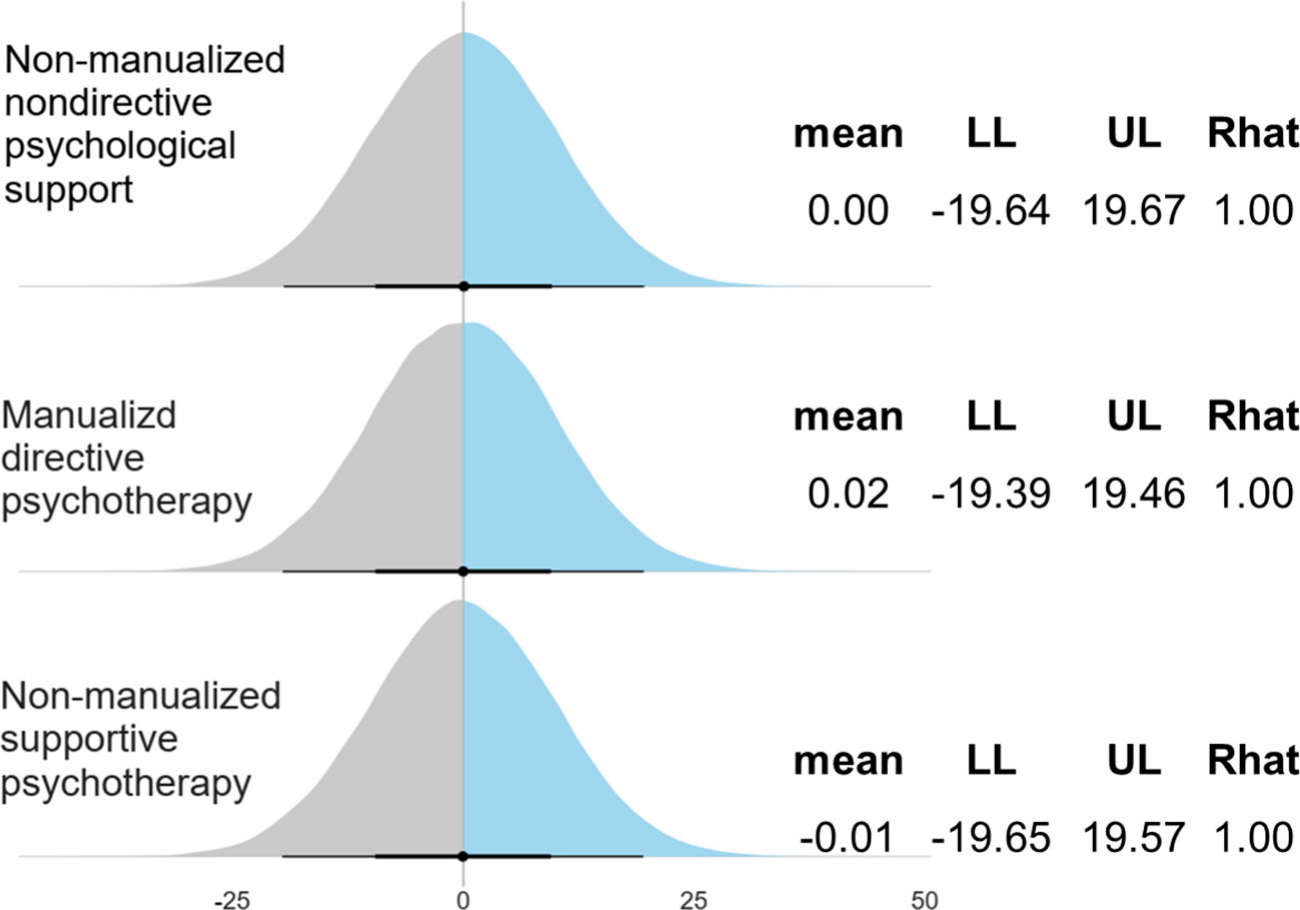
Study characteristics and models of psychological approaches. RCT, randomised controlled trial; Pre-Post, pre-post single-arm design; PS, psychological support; PT, psychotherapy; MDPT, manualized directive psychotherapy; MNDPS, manualized nondirective psychological support; NMNDPS, non-manualized nondirective psychological support; NMSPT, non-manualized supportive psychotherapy. Note: Dose: total dose (mg).

There were ten eligible studies (n=515) that examined the antidepressant effects of psilocybin on adult patients with clinically diagnosed depression. The mean age was 44.0 (6.7) years with 62.1% of female patients. **Figure 1** illustrates the demographic and clinical characteristics of the included studies. These ten trials were conducted between 2011 and 2023. Seven studies recruited adult patients with MDD, and three included mixed conditions (e.g., life-threatening cancer with MDD, generalized anxiety disorder, or persistent depressive disorders). There were seven RCTs, and three pre-post single-arm open-label trials. Six studies used non-manualized psychological approaches and four used manualized ones. Finally, the psychological protocols could be categorized into four types: (i) manualized directive psychotherapy (k=1); (ii) manualized nondirective psychological support (k=3), (iii) non-manualized nondirective psychological support (k=5); and (iv) non-manualized supportive psychotherapy (k=1).

**Table 1** shows the details of the psychological approaches among the ten studies. The components of the manualized directive psychotherapy included supportive, cognitive-behavioral, existentially oriented, and psychoanalytic dimension (17). The manual was based on the guide by Grof (1977): The human encounter with death (21). For the manualized nondirective psychological support, the manuals were based on the following guides (22, 23): (i). Studerus 2012: Prediction of psilocybin response in healthy volunteers. (ii). Tai 2021: Development and evaluation of a therapist training program for psilocybin therapy for treatment-resistant depression in clinical research. Only one study employed supportive psychotherapy (4); however, the content of the supportive psychotherapy resembled that of the other two studies with nondirective psychological support (3, 16). All of these three studies were based on the guide (24): (i). Johnson 2008: Human Hallucinogen research: guidelines for safety.

**Table 1:** Protocols of psychological support or psychotherapy with psilocybin treatment.

Country	Author	Design	Condition	Model	Therapist	Preparation	Integration	As needed Tranquilizer
USA	Grob et al. (2011) (15)	DBPC RCT for inpatient	Advanced cancer with GAD, acute stress disorder, or adjustment disorder	Nondirective psychological support with no manual specified	No information	No information	Discussion of the subjective aesthetic, cognitive, affective, and psychospiritual experiences they had during the session	No information
UK	Carhart-Harris et al. (2016) (3)	OLPP for outpatient	TRD	Nondirective psychological support with no manual specified; Ref: Johnson, 2008; Human Hallucinogen research: guidelines for safety	2 psychiatrists	Preparation: trust, relationship, expectance Support (Acute and peri-acute): empathetic listening and reassurance	Non-judgmental listening to the patient’s testimony after his/her experience and may occasionally feature some interpretation regarding the content of the experience and its potential meaning, as well as advice regarding maintaining and cultivating positive changes in outlook and lifestyle. By telephone the day after low-dose administration; in person the day after the high-dose administration; another visit 1 week after the high-dose administration	Allowable
USA	Griffiths et al. (2016) (16)	DBPC RCT for outpatient	Life-threatening cancer with MDD, GAD, dysthymia, or adjustment disorder	Nondirective psychological support; Ref: Johnson, 2008; Human Hallucinogen research: guidelines for safety	Trained by an experienced psychologist	Discussion of meaningful aspects of the participant’s life, served to establish rapport	During sessions, monitors were nondirective and supportive, and they encouraged participants to “trust, let go and be open” to the experience. Meetings after sessions generally focused on novel thoughts and feelings that arose during sessions	Allowable
USA	Ross et al. (2016) (17)	DBPC RCT for outpatient	Life-threatening cancer with MDD, GAD, or adjustment disorder	Preparatory psychotherapy, medication dosing sessions, and post-dosing integrative psychotherapy with specified manual (component: supportive, cognitive-behavioral, existentially oriented, and psycho-dynamic/ psychoanalytic); Ref: Grof, 1997; The human encounter with death	6 psychiatrists, 2 psychologists, 4 social workers, 1 nurse, 2 master-level counselors	Reviewing the purpose and intention, establishing rapport and trust, and reviewing the life histories (three psychotherapeutic preparation sessions)	Therapists were available for psychological and medical support during the sessions. Participants were encouraged to bring in items of personal significance and meaning and to direct attention to internal experience after psilocybin administration. Towards the end of the dosing session, participants were encouraged to discuss the entirety of their subjective experience with the treatment team to consolidate the memory of the experience (especially given that these experiences can often be ephemeral and difficult to recall in detail, not dissimilar from dream states) and to begin the process of post-integrative psychotherapy. This was akin to doing psychoanalytic psychotherapy with a patient in a type of waking dream-like state. Following each treatment session, three 2-hour psychotherapy sessions were conducted to further consolidate the memory of the experience and to continue the process of psychological integration.	Allowable
USA	Davis et al. (2021) (4)	RCT (delayed controlled) for outpatient	MDD	Supportive psychotherapy Ref: Johnson, 2008; Human Hallucinogen research: guidelines for safety	Facilita2tors with varying educational level (i.e., bachelor’s, master’s, doctorate, and medical degrees) and professional disciplines (e.g., social work, psychology, and psychiatry)	Developing rapport and trust; reviewing the life histories and current situation; discussing intentions and expectations	The participant will meet with the session monitor(s) one day and one week after each session and four weeks after the second session to support integration of session-day experiences. As with any acute, intense positive or negative emotional experience, participants often feel the need for, and seem to benefit from, additional time for reflecting on the novel thoughts and feelings that may have arisen in the session. Given the potentially intense and unusual psychological nature of hallucinogen effects, the volunteer may have difficulty discussing the experience with others in her or his life. Because the monitors were present during the session when the hallucinogen effects were experienced and have knowledge of a broad range of reported phenomena during drug action, the volunteer may feel more comfortable discussing her or his experiences with the monitors than with others. This follow up contact also allows the assessment of any potentially persisting adverse effects, including perceptual abnormalities.	No information
USA	Goodwin et al. (2022) (5)	DBPC RCT for outpatient	TRD	Nondirective psychological support; manualized; Ref: Tai, 2021; Development and Evaluation of a Therapist Training Program for Psilocybin Therapy for Treatment-Resistant Depression in Clinical Research	Two therapists (master’s-level practitioners, nurses, diploma-level cognitive behavioral therapists, or doctorate-level mental health Specialists)	Two sessions for building trust, receiving psychoeducation, and preparing for the psychedelic experience	To support participants in deriving their own insights and solutions from the experience with psilocybin. Therapists were advised to remain open and supportive, without active guiding	No information
Switzerland	von Rotz et al. (2023) (6)	DBPC RCT for outpatient	MDD	Nondirective psychological support with no manual specified (psychological counselling)	Train therapists	Psychological safety and well-being, education, setting an intention, building trust, fostering openness and acceptance	Guidance on the integration of the experience including working through challenging emotions as well as facilitating the creation of a meaningful narrative thereof and support for adequate behavioral adaptations in everyday life	No information
USA	Raison (2023) (18)	DBPC RCT for outpatient	MDD	Nondirective psychological support with manual specified; Set and Setting [SaS] protocol; Usona Facilitator Training Manual and in the study manual of procedures; Ref: Studerus, 2012; Prediction of psilocybin response in healthy volunteers.	Clinical facilitators	To build rapport and therapeutic alliance for navigating the dosing session.	Three integration sessions with details provided in the Usona Facilitator Training Manual and in the study manual of procedures (MoP)	No information
USA	Goodwin (2023) (19)	OLPP for outpatient	TRD	Nondirective psychological support; manualized; Ref: Tai, 2021; Development and Evaluation of a Therapist Training Program for Psilocybin Therapy for Treatment-Resistant Depression in Clinical Research	Mental health professionals with relevant experience, training, and licensure	Building trust with the participant, explaining the trial design and procedures, providing psychoeducation, and helping to prepare the participant for the psilocybin experience	Participants completed an integration session with their lead therapist who encouraged them to derive their own solutions and insights from the psilocybin experience	No information
USA	Aaronson et al. (2023) (20)	OLPP for outpatient	TRD in Bipolar II disorder	Nondirective psychological support in participants currently receiving psychotherapy	A specially trained lead therapist from among a team of doctoral-level psychologists	To build therapeutic rapport, receive psychoeducation, and prepare for the psychedelic experience	Integration sessions occurred during the day after and first and second week of follow-up. These sessions were nondirective and were meant to support the participant in consolidating insights from their psychedelic experience.	No information

DBPC, double-blind placebo-controlled; GAD, generalized anxiety disorder; MDD, major depressive disorder; OLPP, open-label pre-post single-arm; RCT, randomised controlled trial; Ref, reference; TRD, treatment-resistant depression.

### Risk of bias of the included studies

Three studies (3/10) was rated as having a high overall ROB (**Supplementary Figure 2**). The percentages of studies with high, some concerns, and low ROB for the individual items of psychedelic trials were as follows: 0%, 14.3%, and 85.7% for randomization; 0%, 14.3%, and 85.7% for deviations from intended interventions; 0%, 30.0%, and 70.0% for missing outcome data; 30.0%, 30.0%, and 40.0% for measurements of outcomes; 0%, 0%, and 100% for selection of reported results.

### Primary outcome: depressive symptoms

**Figure 2:**
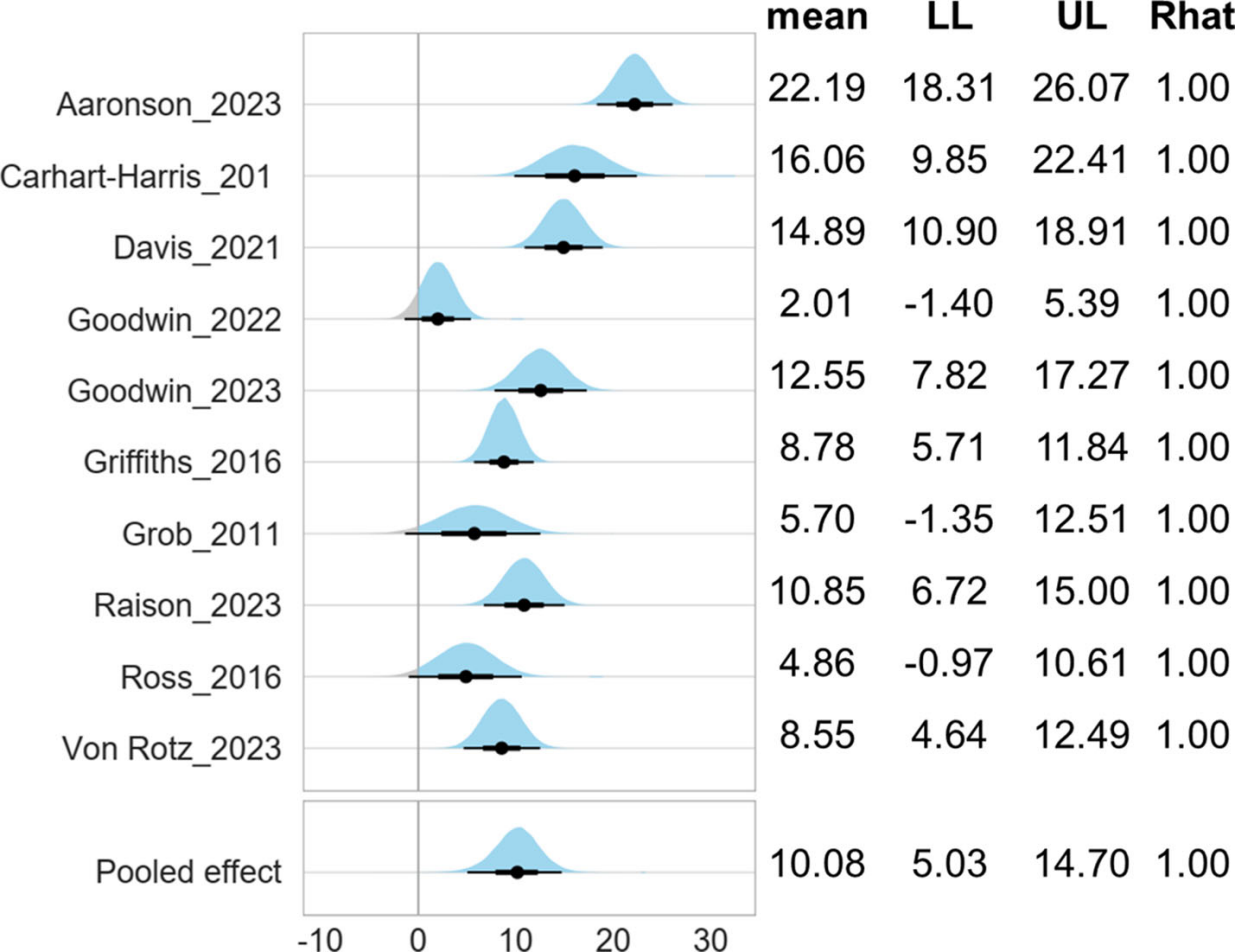
Forest plot of psilocybin-assisted therapy for patients with depressive symptoms. LL, lower limit of 95% credible interval; UL, upper limit of 95% credible interval. Note 1: The mean score was measured by 17-Item Hamilton Depression Rating Scale. Note 2: The Gelman and Rubin convergence diagnostic, or Rhat, approaching 1 indicates that multiple independent Markov Chain Monte Carlo (MCMC) chains have converged to similar distributions, suggesting that the simulation has reached a stable and reliable result.

**Figure 2** shows the pooled effect size of psilocybin-assisted therapy with a mean difference of 10.08 (5.03-14.70).

### Subgroup analysis

**Figure 3:**
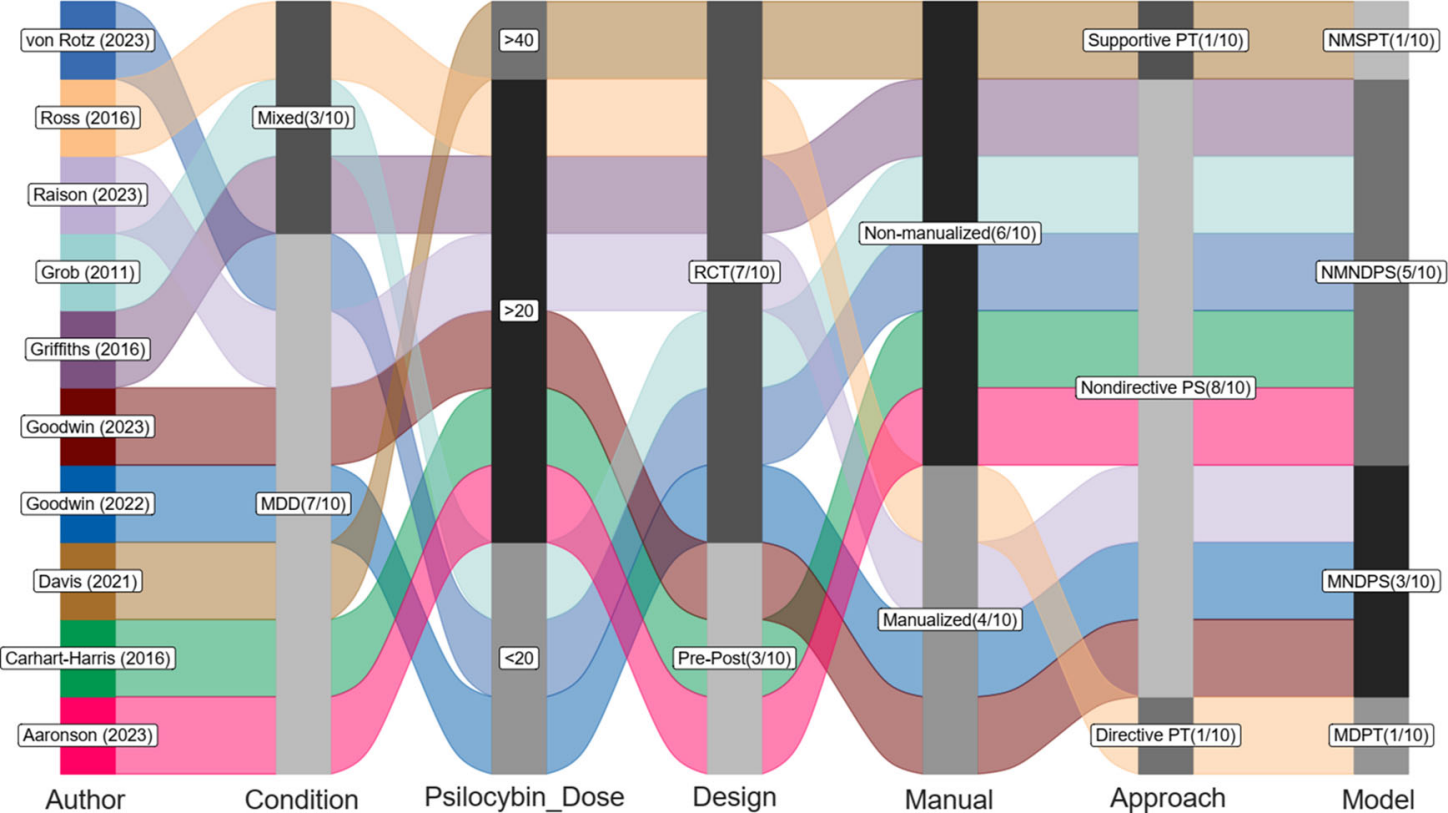
Comparisons between different psychological approaches (reference: manualized nondirective psychological support). LL, lower limit of 95% credible interval; UL, upper limit of 95% credible interval. Note 1: The mean score was measured by 17-Item Hamilton Depression Rating Scale. Note 2: The Gelman and Rubin convergence diagnostic, or Rhat, approaching 1 indicates that multiple independent Markov Chain Monte Carlo (MCMC) chains have converged to similar distributions, suggesting that the simulation has reached a stable and reliable result. Note 3: Compared with manualized nondirective psychological support with adjustment of baseline severity, age, female proportion, dose of psilocybin.

**Figure 3** shows the comparable effectiveness of different psychological approaches. Compared with manualized nondirective psychological support, the other three psychological approaches did not differ significantly, with adjustment of baseline severity, age, female proportion, dose of psilocybin.

### Publication bias

**Figure 4:**
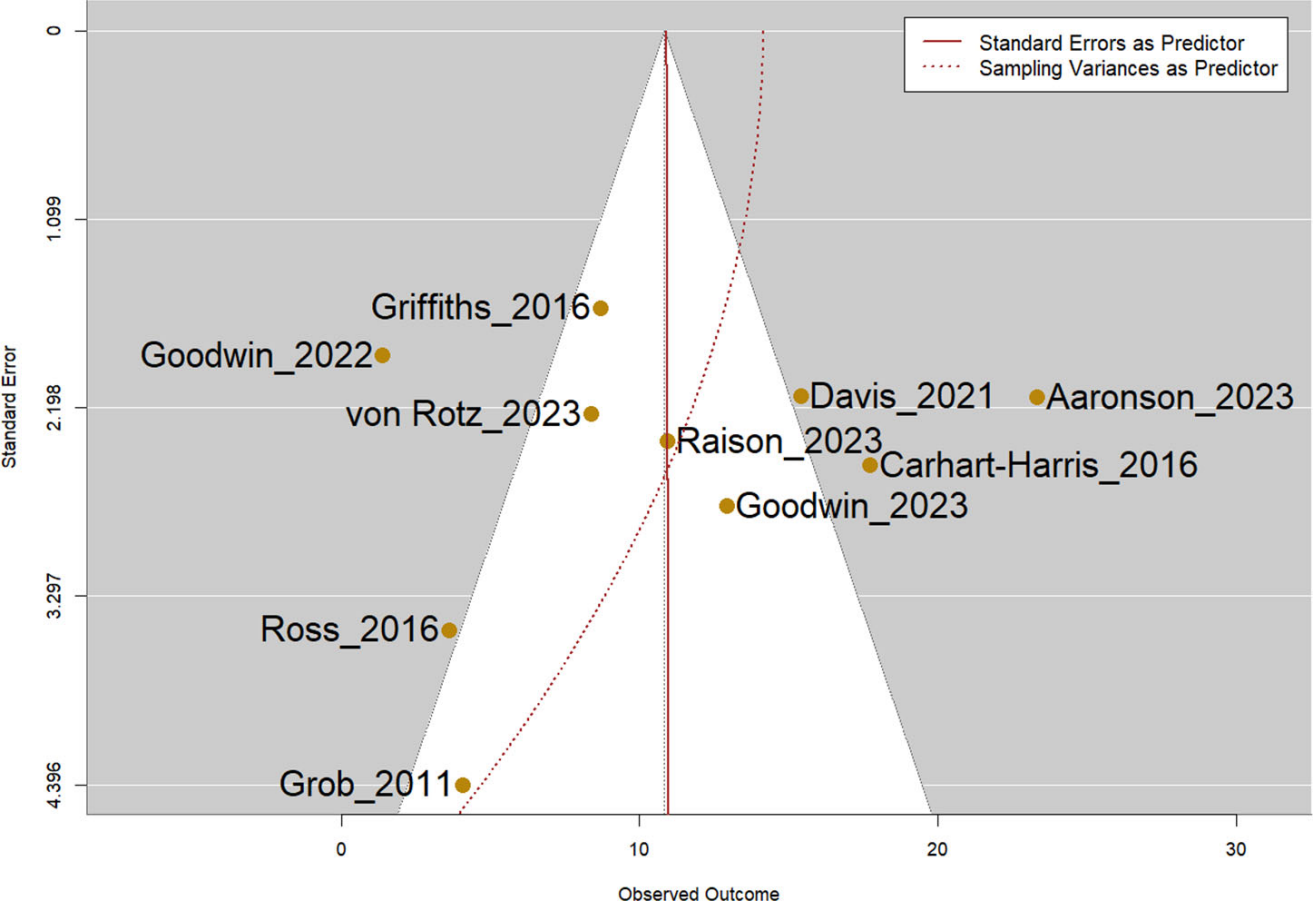
Funnel plot of all included studies. Note: Egger’s test was not statistically significant (p=0.51).

Visual inspection of the funnel plot did not show apparent publication bias, and the Egger’s test was not statistically significant (p=0.51, **Figure 4**).

## Discussion

Psilocybin-assisted therapy is an emerging therapeutic approach that combines psilocybin, a classic psychedelic drug, with psychological support or psychotherapy to address MDD or TRD. The integration of psychedelics and psychotherapy may leverage distinctive interplay between pharmacological, neural, and psychological effects, providing new promising avenues in the challenging landscape of MDD (12). However, the psychological approaches in previous psilocybin trials were not applied in a standardized way (9). The complex psychotherapeutic components complicate the protocols of psilocybin-assisted therapy. As expected, it is difficult to establish a rigid set of standardized psychological support or psychotherapy in psilocybin-assisted therapy even though psilocybin has been approved for TRD in clinical practice in Australia (8). Based on our systematic review, we found that most of the included psilocybin trials were combined with nondirective psychological support without specific manuals, and three used manualized nondirective psychological support. There was one study using non-manualized supportive psychotherapy (4), but the content was similar to those of nondirective psychological support. Only one study using manualized directive psychotherapy (17). However, with adjustment of baseline depression severity, age, female proportion, dose of psilocybin, there were not significant difference between these psychological protocols in association with the efficacy of psilocybin-assisted therapy.

The therapeutic mechanisms of psilocybin for MDD remains incompletely understood. It is believed that pharmacological, neural, and psychological underpinning contributed to its efficacy on MDD (25). At the pharmacological level, psilocybin primarily acts on the receptor with indirect effects on the dopaminergic and glutamatergic systems, leading to gene expression, increased neuroplasticity, and anti-inflammation. At the pharmacological level, psilocybin primarily activates the 5-HT_2A_ (5-hydroxytryptamine) receptor and indirectly modulates the dopaminergic and glutamatergic systems. These interactions results in gene expression, heightened neuroplasticity, and anti-inflammatory responses (25). At the neural level, the response to psilocybin correlated with decreases in large-scale network modularity, suggesting that psilocybin’s antidepressant action may depend on a global increase in how different brain networks are integrated (26). At the psychological level, psilocybin is administered with psychological support or psychotherapy. When delivered in a secure and therapeutic environment, along with psychological assistance, participants often describe transformative experiences marked by profound shifts in values, beliefs, and perspectives. These changes can result in enhanced subjective well-being, increased openness, and improved cognitive flexibility (25, 27).

For the potential clinical application, psilocybin-assisted psychotherapy has sparked debate among psychiatrists, psychologists, neuroscientists, and advocates of psychedelics. This has led to the emergence of two factions: psilocybin therapy as a pure biological intervention and psilocybin as a catalyst for psychotherapy (28). Clearly, both sides are supported by equally valid arguments. On one hand, proponents of psilocybin-assisted therapy as a form of psychotherapy emphasize the following points. First, the effects of psychedelics have been observed to be highly dependent on contextual factors (ie, set and setting) (29). The psychopathology may exacerbate if the psychedelic experiences develop in adverse environments. Second, the nondirective psychological support is still a process-directive psychotherapeutic procedure (23). The authors of the therapist training program acknowledge that their psychological support framework incorporates elements from cognitive-behavioral therapy, mindfulness, Acceptance and Commitment Therapy, and Gestalt therapy model of focusing (23).

On the other hand, the effects of psilocybin have been argued to result from the drug itself and not from psychological interventions (30). In a RCT involving 233 patients with TRD, a dose-response relationship was observed between psilocybin dosage and depression improvement (5). Specifically, a separation of 25 mg, 10 mg, and 1mg psilocybin doses was identified. The psychological support was consistently administered across all three groups, indicating that the efficacy of psilocybin was largely contingent on mechanisms (5). Besides, a recent study found that psychedelics can function as positive allosteric modulators, fostering neuroplasticity through direct binding to the BDNF (brain-derived neurotrophic factor) receptor TrkB (tropomyosin receptor kinase) (31). This means psychedelics may exert antidepressant effect without inducing hallucinogenic effects, further supporting that psilocybin may produce therapeutic effects without experiencing psychedelic experiences. The United States FDA has published a new draft guidance to provide general considerations to sponsors developing psychedelic drugs for treatment of medical conditions (https://www.fda.gov/news-events/press-announcements/fda-issues-first-draft-guidance-clinical-trials-psychedelic-drugs, last access on Feb. 4 2024). In this guidance, it is recommended to employ a factorial design in future RCTs to distinguish and assess the individual contributions of drug and psychotherapy to any observed treatment response. Although our findings indicate that different psychotherapies do not show significant differences in antidepressant efficacy with psilocybin, these psychotherapies may still have some therapeutic effects. Through the factorial design RCTs, clinicians might build a standardized treatment protocol and a standardized tutor training program to ensure the effectiveness and safety of the psilocybin-assisted treatment.

Our study still has several limitations. First, some studies did not specify which guidance they followed, and we only could provide the information from the published papers (15, 20). Second, we did not include psilocybin studies addressing other psychiatric conditions such as substance use disorder. We also did not include other psychedelic-assisted therapy such as 3,4-methylenedioxymethamphetamine. However, focusing on a single psychedelic (psilocybin) and a single condition (clinically diagnosed depression) allows for a better understanding of the inconsistencies and diversity in protocols used simultaneously.

## Conclusion

Over the past decades, accumulative evidence from clinical trials reveals promising results of psychedelic drugs in the treatment of various psychiatric disorders. Our study clearly showed diverse protocols of psychological support or psychotherapy with psilocybin treatment. Nondirective client-led therapy was the most commonly employed, and most of these studies used non-manualized protocols. The establishment of a consistent and standardized protocol for psychological strategies might be crucial for the development of a training program among clinical practitioners. However, we did not find significant difference in the association with the antidepressant efficacy among different psychological protocols. In the future, standardized psychological protocols and standardized tutor training programs should be established to ensure the quality of treatment. Furthermore, longitudinal studies or follow-up designs of RCTs are needed to assess the long-term effects of psilocybin-assisted therapy. These suggestions would provide a clearer roadmap for advancing the field.

## Data Availability

The original contributions presented in the study are included in the article/**Supplementary Material**. Further inquiries can be directed to the corresponding authors.

## References

[1] Marwaha S Palmer E Suppes T Cons E Young AH Upthegrove R . Novel and emerging treatments for major depression. Lancet. (2023) 401:141–53. doi: 10.1016/S0140-6736(22)02080-3 36535295

[2] GBD 2019 Diseases and Injuries Collaborators . Global burden of 369 diseases and injuries in 204 countries and territories, 1990-2019: a systematic analysis for the Global Burden of Disease Study 2019. Lancet. (2020) 396:1204–22. doi: 10.1016/S0140-6736(20)30925-9 PMC7567026 33069326

[3] Carhart-Harris RL Bolstridge M Rucker J Day CM Erritzoe D Kaelen M . Psilocybin with psychological support for treatment-resistant depression: an open-label feasibility study. Lancet Psychiatry. (2016) 3:619–27. doi: 10.1016/S2215-0366(16)30065-7 27210031

[4] Davis AK Barrett FS May DG Cosimano MP Sepeda ND Johnson MW . Effects of psilocybin-assisted therapy on major depressive disorder: A randomized clinical trial. JAMA Psychiatry. (2021) 78:481–9. doi: 10.1001/jamapsychiatry.2020.3285 PMC7643046 33146667

[5] Goodwin GM Aaronson ST Alvarez O Arden PC Baker A Bennett JC . Single-dose psilocybin for a treatment-resistant episode of major depression. N Engl J Med. (2022) 387:1637–48. doi: 10.1056/NEJMoa2206443 36322843

[6] von Rotz R Schindowski EM Jungwirth J Schuldt A Rieser NM Zahoranszky K . Single-dose psilocybin-assisted therapy in major depressive disorder: a placebo-controlled, double-blind, randomized clinical trial. eClinicalMedicine. (2023) 56:101809. doi: 10.1016/j.eclinm.2022.101809 PMC9830149 36636296

[7] Heal DJ Smith SL Belouin SJ Henningfield JE . Psychedelics: threshold of a therapeutic revolution. Neuropharmacology. (2023) 236:109610. doi: 10.1016/j.neuropharm.2023.109610 37247807

[8] Nogrady B . Australia’s approval of MDMA and psilocybin for PTSD and depression is premature, say critics. BMJ. (2023) 382:p1599. doi: 10.1136/bmj.p1599 37433614

[9] Butlen-Ducuing F McCulloch DEW Haberkamp M Mattila T Bałkowiec-Iskra E Aislaitner G . The therapeutic potential of psychedelics: the European regulatory perspective. Lancet. (2023) 401:714–6. doi: 10.1016/S0140-6736(23)00264-7 36780909

[10] Sloshower J Skosnik PD Safi-Aghdam H Pathania S Syed S Pittman B . Psilocybin-assisted therapy for major depressive disorder: An exploratory placebo-controlled, fixed-order trial. J Psychopharmacol. (2023) 37:698–706. doi: 10.1177/02698811231154852 36938991

[11] Page MJ McKenzie JE Bossuyt PM Boutron I Hoffmann TC Mulrow CD . The PRISMA 2020 statement: an updated guideline for reporting systematic reviews. BMJ. (2021) 372:n71. doi: 10.1136/bmj.n71 33782057 PMC8005924

[12] Ko K Kopra EI Cleare AJ Rucker JJ . Psychedelic therapy for depressive symptoms: A systematic review and meta-analysis. J Affect Disord. (2023) 322:194–204. doi: 10.1016/j.jad.2022.09.168 36209780

[13] Sterne JAC Savovic J Page MJ Elbers RG Blencowe NS Boutron I . RoB 2: a revised tool for assessing risk of bias in randomized trials. BMJ. (2019) 366:l4898. doi: 10.1136/bmj.l4898 31462531

[14] Phillippo DM . multinma: Bayesian network meta-analysis of individual and aggregate data. Bristol, England: University of Bristol (2020). doi: 10.32614/CRAN.packages

[15] Grob CS Danforth AL Chopra GS Hagerty M McKay CR Halberstadt AL . Pilot study of psilocybin treatment for anxiety in patients with advanced-stage cancer. Arch Gen Psychiatry. (2011) 68:71–8. doi: 10.1001/archgenpsychiatry.2010.116 20819978

[16] Griffiths RR Johnson MW Carducci MA Umbricht A Richards WA Richards BD . Psilocybin produces substantial and sustained decreases in depression and anxiety in patients with life-threatening cancer: A randomized double-blind trial. J Psychopharmacol. (2016) 30:1181–97. doi: 10.1177/0269881116675513 PMC5367557 27909165

[17] Ross S Bossis A Guss J Agin-Liebes G Malone T Cohen B . Rapid and sustained symptom reduction following psilocybin treatment for anxiety and depression in patients with life-threatening cancer: a randomized controlled trial. J Psychopharmacol. (2016) 30:1165–80. doi: 10.1177/0269881116675512 PMC5367551 27909164

[18] Raison CL Sanacora G Woolley J Heinzerling K Dunlop BW Brown RT . Single-dose psilocybin treatment for major depressive disorder: a randomized clinical trial. JAMA. (2023) 330(9):843–853. doi: 10.1001/jama.2023.14530 PMC10472268 37651119

[19] Goodwin GM Croal M Feifel D Kelly JR Marwood SM Mistry S . Psilocybin for treatment resistant depression in patients taking a concomitant SSRI medication. Neuropsychopharmacology. (2023) 48(10):1492–99. doi: 10.1038/s41386-023-01648-7 PMC10425429 37443386

[20] Aaronson ST van der Vaart A Miller T LaPratt J Swartz K Shoultz A . Single-dose synthetic psilocybin with psychotherapy for treatment-resistant bipolar type II major depressive episodes: A nonrandomized controlled trial. JAMA Psychiatry. (2023) 81:552–62. doi: 10.1001/jamapsychiatry.2023.4685 PMC10701666 38055270

[21] Grof S Halifax J . The human encounter with death. New York: EP Dutton (1977).

[22] Studerus E Gamma A Kometer M Vollenweider FX . Prediction of psilocybin response in healthy volunteers. PloS One. (2012) 7:e30800. doi: 10.1371/journal.pone.0030800 22363492 PMC3281871

[23] Tai SJ Nielson EM Lennard-Jones M Johanna Ajantaival RL Winzer R Richards WA . Development and evaluation of a therapist training program for psilocybin therapy for treatment-resistant depression in clinical research. Front Psychiatry. (2021) 12:586682. doi: 10.3389/fpsyt.2021.586682 33643087 PMC7908919

[24] Johnson M Richards W Griffiths R . Human hallucinogen research: guidelines for safety. J Psychopharmacol. (2008) 22:603–20. doi: 10.1177/0269881108093587 PMC3056407 18593734

[25] van Elk M Yaden DB . Pharmacological, neural, and psychological mechanisms underlying psychedelics: A critical review. Neurosci Biobehav Rev. (2022) 140:104793. doi: 10.1016/j.neubiorev.2022.104793 35878791

[26] Daws RE Timmermann C Giribaldi B Sexton JD Wall MB Erritzoe D . Increased global integration in the brain after psilocybin therapy for depression. Nat Med. (2022) 28:844–51. doi: 10.1038/s41591-022-01744-z 35411074

[27] Doss MK Považan M Rosenberg MD Sepeda ND Davis AK Finan PH . Psilocybin therapy increases cognitive and neural flexibility in patients with major depressive disorder. Transl Psychiatry. (2021) 11:574. doi: 10.1038/s41398-021-01706-y 34750350 PMC8575795

[28] Gründer G Brand M Mertens LJ Jungaberle H Kärtner L Scharf DJ . Treatment with psychedelics is psychotherapy: beyond reductionism. Lancet Psychiatry. (2024) 11:231–6. doi: 10.1016/S2215-0366(23)00363-2 38101439

[29] Carhart-Harris RL Roseman L Haijen E Erritzoe D Watts R Branchi I . Psychedelics and the essential importance of context. J Psychopharmacol. (2018) 32:725–31. doi: 10.1177/0269881118754710 29446697

[30] Goodwin GM Malievskaia E Fonzo GA Nemeroff CB . Must psilocybin always “Assist psychotherapy”? Am J Psychiatry. (2024) 181:20–5. doi: 10.1176/appi.ajp.20221043 37434509

[31] Moliner R Girych M Brunello CA Kovaleva V Biojone C Enkavi G . Psychedelics promote plasticity by directly binding to BDNF receptor TrkB. Nat Neurosci. (2023) 26:1032–41. doi: 10.1038/s41593-023-01316-5 PMC10244169 37280397

