# Supplementary material for: The association between diverse psychological protocols and the efficacy of psilocybin-assisted therapy for clinical depressive symptoms: a Bayesian meta-analysis
Source: Front Psychiatry. 2024 Aug 13;15:1439347. doi: 10.3389/fpsyt.2024.1439347 (PMC11347349; doi:10.3389/fpsyt.2024.1439347)

**The association between diverse psychological protocols and the efficacy of psilocybin-assisted therapy for clinical depressive symptoms: a Bayesian meta-analysis**

eFigure 1. Study flow chart

eFigure 2. Risk of bias plot

Appendix 1. The PRISMA

Appendix 2. The complete search strategies

Appendix 3. Reasons for exclusion

Appendix 4. Prior settings and results of convergence

**eFigure 1. Study flow chart**

**Identification of studies via databases and registers**

Records identified from

Embase (n= 78)

MEDLINE (n= 320)

CENTRAL (n= 149)

PsycINFO (n= 169)

ClinicalTrials.gov (n= 52)

World Health Organization

(ICTRP) (n= 53):

Databases (n =821)

Records removed *before screening*: Duplicate records removed (n = 172)

**Identification**

Records excluded title and abstract (n = 625)

Records screened

(n = 649)

**Screening**

Reports not retrieved

(n = 0)

Reports sought for retrieval

(n = 24)

Reports excluded:

Conference abstract, protocol, comment, or review (n =3)

Duplicated database (n =8)

No outcome of interest (n =1)

Head-to-head study or fixed-order study (n=2)

Reports assessed for eligibility

(n =24)

Studies included in review (n =10)

Reports of included studies (n =10)

**Included**

*From:*  Page MJ, McKenzie JE, Bossuyt PM, Boutron I, Hoffmann TC, Mulrow CD, et al. The PRISMA 2020 statement: an updated guideline for reporting systematic reviews. BMJ 2021;372:n71. doi: 10.1136/bmj.n71

**eFigure 2. Risk of bias plot**


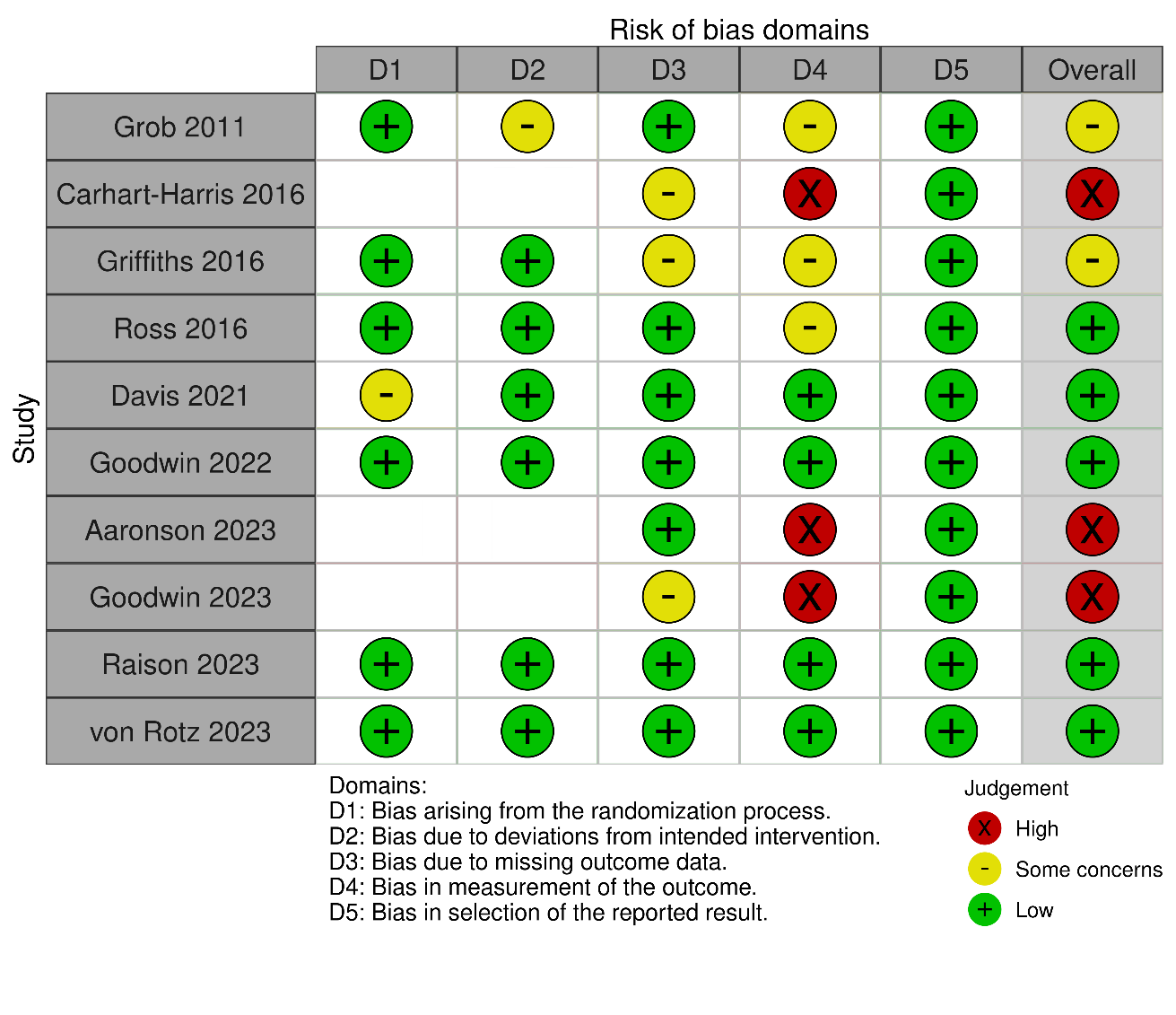


**Appendix 1**. Checklist of PRISMA guideline

| **Section and Topic** | **Item #** | **Checklist item** | **Location where item is reported** |
| --- | --- | --- | --- |
| **TITLE** | | |  |
| Title | 1 | Identify the report as a systematic review. | title |
| **ABSTRACT** | | |  |
| Abstract | 2 | See the PRISMA 2020 for Abstracts checklist. | Abstract section |
| **INTRODUCTION** | | |  |
| Rationale | 3 | Describe the rationale for the review in the context of existing knowledge. | 1st -3rd paragraph of introduction |
| Objectives | 4 | Provide an explicit statement of the objective(s) or question(s) the review addresses. | 4th paragraph of introduction |
| **METHODS** | | |  |
| Eligibility criteria | 5 | Specify the inclusion and exclusion criteria for the review and how studies were grouped for the syntheses. | paragraph of " Eligibility Criteria " of methods |
| Information sources | 6 | Specify all databases, registers, websites, organisations, reference lists and other sources searched or consulted to identify studies. Specify the date when each source was last searched or consulted. | paragraph of " Data Sources and Search " and “Eligibility Criteria” of methods |
| Search strategy | 7 | Present the full search strategies for all databases, registers and websites, including any filters and limits used. | Appendix 2 |
| Selection process | 8 | Specify the methods used to decide whether a study met the inclusion criteria of the review, including how many reviewers screened each record and each report retrieved, whether they worked independently, and if applicable, details of automation tools used in the process. | paragraph of " Data Sources and Search " and “Eligibility Criteria” of methods |
| Data collection process | 9 | Specify the methods used to collect data from reports, including how many reviewers collected data from each report, whether they worked independently, any processes for obtaining or confirming data from study investigators, and if applicable, details of automation tools used in the process. | paragraph of " Definitions and Data Extraction" of methods |
| Data items | 10a | List and define all outcomes for which data were sought. Specify whether all results that were compatible with each outcome domain in each study were sought (e.g. for all measures, time points, analyses), and if not, the methods used to decide which results to collect. | paragraph of " Definitions and Data Extraction" of methods |
|  | 10b | List and define all other variables for which data were sought (e.g. participant and intervention characteristics, funding sources). Describe any assumptions made about any missing or unclear information. | paragraph of " Definitions and Data Extraction" of methods |
| Study risk of bias assessment | 11 | Specify the methods used to assess risk of bias in the included studies, including details of the tool(s) used, how many reviewers assessed each study and whether they worked independently, and if applicable, details of automation tools used in the process. | paragraph of " Risk of bias assessment" of methods |
| Effect measures | 12 | Specify for each outcome the effect measure(s) (e.g. risk ratio, mean difference) used in the synthesis or presentation of results. | paragraph of " Statistical Analysis" of methods |
| Synthesis methods | 13a | Describe the processes used to decide which studies were eligible for each synthesis (e.g. tabulating the study intervention characteristics and comparing against the planned groups for each synthesis (item #5)). | paragraph of " Statistical Analysis" of methods |
|  | 13b | Describe any methods required to prepare the data for presentation or synthesis, such as handling of missing summary statistics, or data conversions. | paragraph of " Statistical Analysis" of methods |
|  | 13c | Describe any methods used to tabulate or visually display results of individual studies and syntheses. | paragraph of " Statistical Analysis" of methods |
|  | 13d | Describe any methods used to synthesize results and provide a rationale for the choice(s). If meta-analysis was performed, describe the model(s), method(s) to identify the presence and extent of statistical heterogeneity, and software package(s) used. | paragraph of " Statistical Analysis" of methods |
|  | 13e | Describe any methods used to explore possible causes of heterogeneity among study results (e.g. subgroup analysis, meta-regression). | paragraph of " Statistical Analysis" of methods |
|  | 13f | Describe any sensitivity analyses conducted to assess robustness of the synthesized results. | paragraph of " Statistical Analysis" of methods |
| Reporting bias assessment | 14 | Describe any methods used to assess risk of bias due to missing results in a synthesis (arising from reporting biases). | Not applicable |
| Certainty assessment | 15 | Describe any methods used to assess certainty (or confidence) in the body of evidence for an outcome. | Not applicable |
| **RESULTS** | | |  |
| Study selection | 16a | Describe the results of the search and selection process, from the number of records identified in the search to the number of studies included in the review, ideally using a flow diagram. | Supplement 4 (PRISMA 2020 flow diagram) |
|  | 16b | Cite studies that might appear to meet the inclusion criteria, but which were excluded, and explain why they were excluded. | Appendix 3 |
| Study characteristics | 17 | Cite each included study and present its characteristics. | Table 1, 1^st^ paragraph of results |
| Risk of bias in studies | 18 | Present assessments of risk of bias for each included study. | paragraph of " Risk of bias of the included studies" of result  eFigure 2 |
| Results of individual studies | 19 | For all outcomes, present, for each study: (a) summary statistics for each group (where appropriate) and (b) an effect estimate and its precision (e.g. confidence/credible interval), ideally using structured tables or plots. | paragraph of “Primary outcome: depressive symptoms” result  Figure 2 |
| Results of syntheses | 20a | For each synthesis, briefly summarise the characteristics and risk of bias among contributing studies. | Figure 2, eFigure 2  paragraph of “Primary outcome: depressive symptoms” and “Risk of bias of the included studies” of result. |
|  | 20b | Present results of all statistical syntheses conducted. If meta-analysis was done, present for each the summary estimate and its precision (e.g. confidence/credible interval) and measures of statistical heterogeneity. If comparing groups, describe the direction of the effect. | paragraph of “Primary outcome: depressive symptoms” result  Figure 2 |
|  | 20c | Present results of all investigations of possible causes of heterogeneity among study results. | Figure 3, paragraph of “Subgroup analysis” |
|  | 20d | Present results of all sensitivity analyses conducted to assess the robustness of the synthesized results. | Figure 3, paragraph of “Subgroup analysis” |
| Reporting biases | 21 | Present assessments of risk of bias due to missing results (arising from reporting biases) for each synthesis assessed. | Not applicable |
| Certainty of evidence | 22 | Present assessments of certainty (or confidence) in the body of evidence for each outcome assessed. | Not applicable |
| **DISCUSSION** | | |  |
| Discussion | 23a | Provide a general interpretation of the results in the context of other evidence. | 1^st^ paragraph of discussion |
|  | 23b | Discuss any limitations of the evidence included in the review. | 5^th^ paragraph of discussion |
|  | 23c | Discuss any limitations of the review processes used. | 5^th^ paragraph of discussion |
|  | 23d | Discuss implications of the results for practice, policy, and future research. | Conclusions section |
| **OTHER INFORMATION** | | |  |
| Registration and protocol | 24a | Provide registration information for the review, including register name and registration number, or state that the review was not registered. | PROSPERO (521995) |
|  | 24b | Indicate where the review protocol can be accessed, or state that a protocol was not prepared. | PROSPERO (521995) |
|  | 24c | Describe and explain any amendments to information provided at registration or in the protocol. | None |
| Support | 25 | Describe sources of financial or non-financial support for the review, and the role of the funders or sponsors in the review. | None |
| Competing interests | 26 | Declare any competing interests of review authors. | None |
| Availability of data, code and other materials | 27 | Report which of the following are publicly available and where they can be found: template data collection forms; data extracted from included studies; data used for all analyses; analytic code; any other materials used in the review. | Not applicable |

**Appendix 2**. The complete search strategies

| Database |
| --- |
| MEDLINE search strategy |
| 1 exp Psilocybin/ or Psilocybin.mp. or magic mushrooms.mp. (1,720)  2 exp Depression/ or Depression.mp. (492,128)  3 depress*.mp. (647,538)  4 2 or 3 (647,538)  5 clinical trial.mp. or exp Clinical Trial/ (1,094,828)  17 1 and 4 and 5 (320) |
| Cochrane Central Register of Controlled Trials (CENTRAL) search strategy |
| #1 MeSH descriptor: [Psilocybin] explode all trees (153)  #2 (Psilocybin):ti,ab,kw (307)  #3 #1 or #2 (345)  #4 MeSH descriptor: [Depression] 1 tree(s) exploded (18679)  #5 (Depression):ti,ab,kw (98,664)  #6 #4 or #5 (98,664)  #7 ("randomized controlled trial" or RCT):ti,ab,kw (669,993)  #8 #3 and #6 and #7 (149) |
| Embase search strategy |
| #1. 'psilocybin'/exp OR psilocybin (3,067)  #2. 'magic mushrooms' (163)  #3. 'depression'/exp OR depression (929,175)  #4. #1 OR #2 (3092)  #5. #3 AND #4 AND clinical trial (78) |
| PsycINFO search strategy |
| S1 psilocybin OR magic mushrooms (906)  S2 depression or depressive disorder or depressive symptoms or major depressive  disorder (418,881)  S3 (S1 AND S2) (289)  S4 (clinical trial AND S3) (169) |
| ClinicalTrials.gov (https://clinicaltrials.gov/) |
| Psilocybin \| Depression (52) |
| World Health Organization (ICTRP) (https://trialsearch.who.int) |
| Psilocybin and Depression (53) |

CENTRAL: Cochrane Central Register of Controlled Trials

**Appendix 3.** Reasons for exclusion

**Duplicated (n=8)**

Barba T, Buehler S, Kettner H, et al. Effects of psilocybin versus escitalopram on rumination and thought suppression in depression. *BJPsych open* 2022;8(5) doi: 10.1192/bjo.2022.565

Goodwin GM, Aaronson ST, Alvarez O, et al. Single-dose psilocybin for a treatment-resistant episode of major depression: impact on patient-reported depression severity, anxiety, function, and quality of life. *Journal of affective disorders* 2023;327:120‐27. doi: 10.1016/j.jad.2023.01.108

Goodwin GM, Croal M, Feifel D, et al. Psilocybin for treatment resistant depression in patients taking a concomitant SSRI medication. *Neuropsychopharmacology* 2023;48(10):1492-99. doi: 10.1038/s41386-023-01648-7

Goodwin GM, Marwood L, Mistry S, et al. Improvement in Depression Symptoms Measured by Montgomery-Åsberg Depression Rating Scale and Quick Inventory of Depressive Symptomatology-Self Rated Items after Randomised Double-blind COMP360 Psilocybin Therapy for Treatment-resistant Depression. *European Psychiatry* 2023;66:S91-S92. doi: 10.1192/j.eurpsy.2023.273

Gukasyan N, Davis AK, Barrett FS, et al. Efficacy and safety of psilocybin-assisted treatment for major depressive disorder: prospective 12-month follow-up. *Journal of psychopharmacology (Oxford, England)* 2022;36(2):151‐58. doi: 10.1177/02698811211073759

Murphy R, Kettner H, Zeifman R, et al. Therapeutic Alliance and Rapport Modulate Responses to Psilocybin Assisted Therapy for Depression. *Frontiers in pharmacology* 2022;12 doi: 10.3389/fphar.2021.788155

Weiss B, Ginige I, Shannon L, et al. Personality change in a trial of psilocybin therapy v. escitalopram treatment for depression. *Psychological medicine* 2023:1‐15. doi: 10.1017/S0033291723001514

Zeifman RJ, Wagner AC, Monson CM, et al. How does psilocybin therapy work? An exploration of experiential avoidance as a putative mechanism of change. *Journal of affective disorders* 2023;334:100‐12. doi: 10.1016/j.jad.2023.04.105

**Outcome not interest (n=1)**

Marschall J, Fejer G, Lempe P, et al. Psilocybin microdosing does not affect emotion-related symptoms and processing: A preregistered field and lab-based study. *Journal of psychopharmacology (Oxford, England)* 2022;36(1):97-113. doi: https://dx.doi.org/10.1177/02698811211050556

**Comment, Conference abstract, protocol, or review (n=3)**

Griffiths R, Barrett F, Darrick M, et al. Psilocybin-assisted treatment of major depressive disorder: results from a randomized trial. *Neuropsychopharmacology* 2019;44:439. doi: 10.1038/s41386-019-0547-9

Husain MI, Blumberger DM, Castle DJ, et al. Psilocybin for treatment-resistant depression without psychedelic effects: study protocol for a 4-week, double-blind, proof-of-concept randomised controlled trial. *BJPsych open* 2023;9(4):e134. doi: 10.1192/bjo.2023.535

Rosenblat J, McIntyre R. Psilocybin Assisted Therapy for Treatment-Resistant Depression: a Phase II, Randomized, Feasibility Study. *Neuropsychopharmacology* 2022;47:203. doi: 10.1038/s41386-022-01484-1

**Head-to-head or Fixed-order studies (n=2)**

Sloshower J, Skosnik PD, Safi-Aghdam H, et al. Psilocybin-assisted therapy for major depressive disorder: An exploratory placebo-controlled, fixed-order trial. J Psychopharmacol. 2023;37(7):698-706. doi:10.1177/02698811231154852

Carhart-Harris R, Giribaldi B, Watts R, et al. Trial of Psilocybin versus Escitalopram for Depression. N Engl J Med. 2021;384(15):1402-1411. doi:10.1056/NEJMoa2032994

**Appendix 4. Prior settings and results of convergence**

**Prior settings**

Prior distribution for the treatment: A Normal prior distribution: location = 0, scale = 10.

Prior distribution for the heterogeneity: A half-Cauchy prior distribution: location = 0, scale = 10.

Prior distribution for the heterogeneity type: sd.

Prior distribution for the regression coefficients: A Normal prior distribution: location = 0, scale = 10.

Prior distribution for the intercept: Null

Prior distribution for the auxiliary parameter: Null

Trace plot for Bayesian meta-analysis


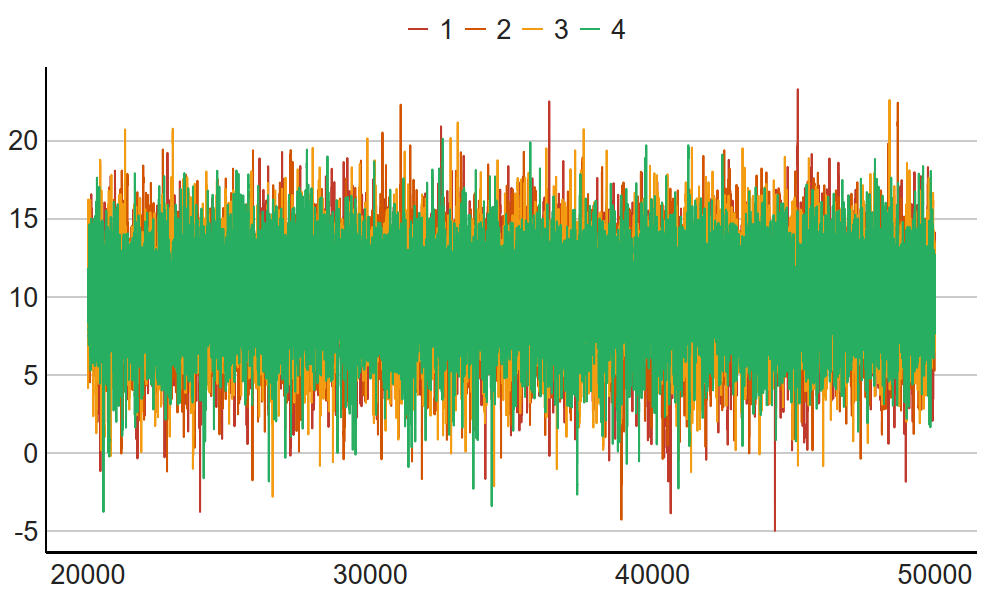


Trace plot for Bayesian meta-regression


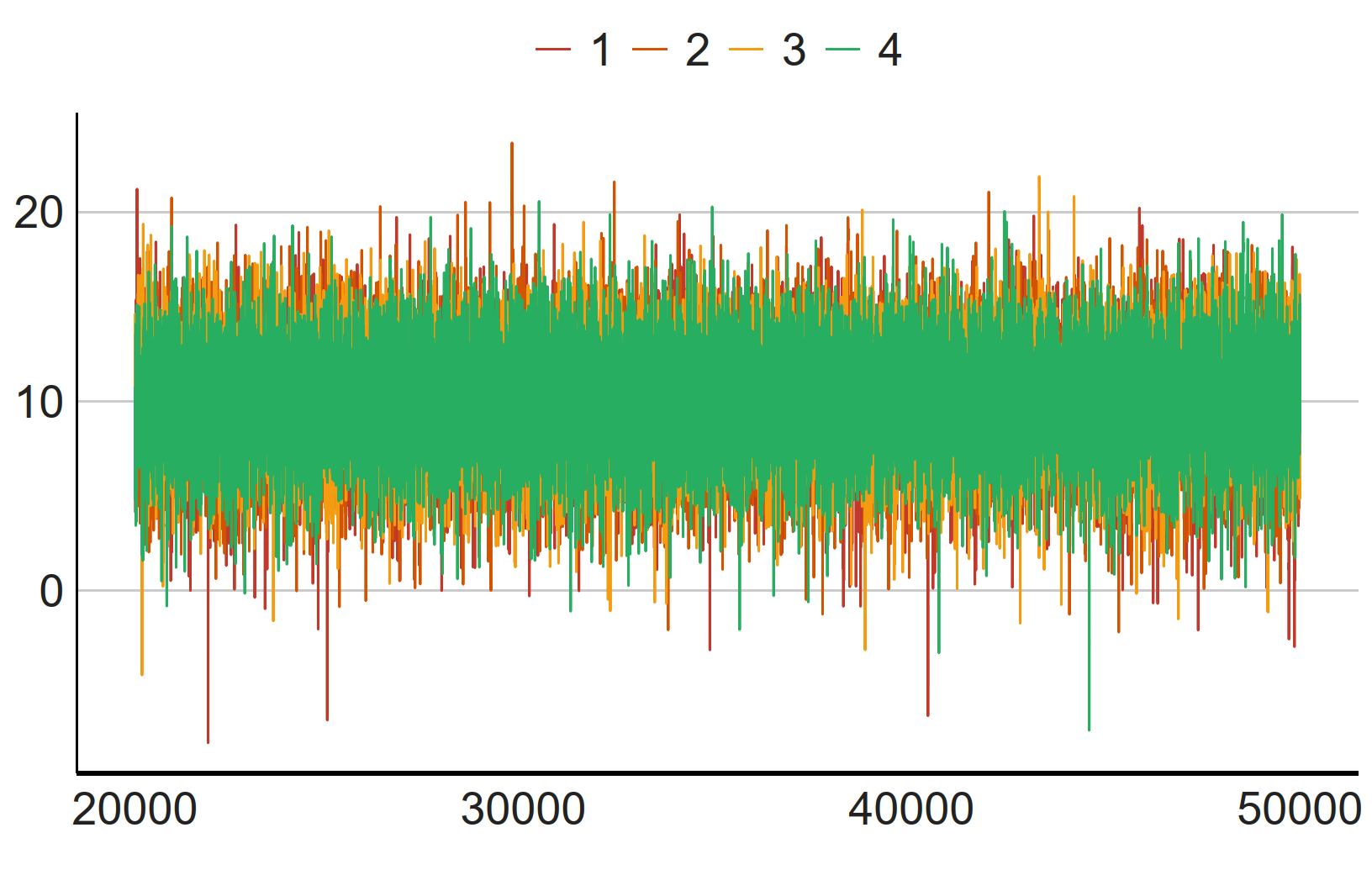

Supplement: Supplementary file 1 [file DataSheet1.docx]
